# Peripheral blood B-cell compartment dysregulation in multidrug-resistant tuberculosis is associated with reduced circulating marginal zone-like B cells

**DOI:** 10.3389/fimmu.2026.1709981

**Published:** 2026-02-10

**Authors:** Mei Liu, Junjie Wen, Qin Gao, Tao Xu, Yuanbo Lan, Lu Meng

**Affiliations:** 1 Department of Tuberculosis, Affiliated Hospital of Zunyi Medical University, Zunyi, Guizhou, China; 2 Guizhou Provincial Key Laboratory of Pathogenesis and Prevention for Common Chronic Diseases, Affiliated Hospital of Zunyi Medical University, Zunyi, Guizhou, China; 3 Guizhou Provincial 2011 Collaborative Innovation Center for Comprehensive Tuberculosis Prevention and Treatment, Affiliated Hospital of Zunyi Medical University, Zunyi, Guizhou, China; 4 Institute of Infection and Health, Fudan University, Shanghai, China; 5 Institute of Science, Fudan University, Shanghai, China; 6 Department of Infectious Diseases, Huashan Hospital, Shanghai, China

**Keywords:** B-cell subsets, flow cytometry, immune dysregulation, marginal zone-like B cells (MZ B), multidrug-resistant tuberculosis

## Abstract

**Objective:**

Multidrug-resistant tuberculosis (MDR-TB) remains a major global health challenge. While T cell-mediated immunity in tuberculosis is well characterized, alterations in circulating B-cell subsets during chronic MDR-TB are less well defined.

**Methods:**

Peripheral blood mononuclear cells (PBMCs) from healthy controls [interferon gamma release assay negative (IGRA^−^)], individuals with latent tuberculosis infection (LTBI; IGRA^+^), and patients with active tuberculosis (ATB) were analyzed using multiparameter flow cytometry panels. Major lymphoid and myeloid populations and detailed B-cell subsets were quantified.

**Results:**

Frequencies of major T-cell and natural killer (NK)-cell populations were broadly similar across groups. In contrast, patients with ATB showed a reduction in total CD19^+^ B cells. Within the B-cell compartment, ATB was characterized by an increased proportion of naïve B cells and a pronounced reduction in antibody-secreting cells (ASCs). Circulating marginal zone-like B cells (MZ B, IgD^+^IgM^+^CD27^+^) were also reduced in ATB compared with non-ATB groups. Receiver operating characteristic (ROC) analysis suggested that reduced MZ B-cell frequency may help discriminate individuals with ATB from those without ATB; however, this observation should be interpreted as exploratory given the cohort size and composition.

**Conclusion:**

MDR-TB is associated with broad perturbations of the peripheral B-cell compartment, including reduced ASCs and decreased circulating MZ B cells. These findings highlight B-cell dysregulation as a feature of active disease and identify MZ B cells as a subset of interest for further investigation rather than as a stand-alone diagnostic marker.

## Introduction

Tuberculosis (TB), caused by the airborne pathogen *Mycobacterium tuberculosis* (*Mtb*), remains a major global health challenge. The emergence of multidrug-resistant TB (MDR-TB), defined as resistance to at least isoniazid and rifampicin, poses a catastrophic threat to global TB control (1). According to World Health Organization reports, TB claimed approximately 1.3 million lives in 2023 alone, surpassing mortality rates of all other single infectious pathogens. This persistent burden underscores the critical need for improved diagnostic and therapeutic strategies.

Following alveolar invasion, *Mtb* induces a spectrum of infection states modulated by host immune competence (2–4). Clinically, TB status is categorized into three distinct states: active TB (ATB), characterized by symptomatic, transmissible infection; latent TB infection [LTBI; interferon gamma release assay-positive (IGRA^+^)], representing asymptomatic microbial persistence; and healthy controls (IGRA^−^). Emerging evidence indicates that circulating immune cell subsets undergo differential modulation during *Mtb* disease progression. While cell-mediated immunity, particularly CD4^+^ T-cell responses, is central to mycobacterial control (5, 6), the complex interplay of multiple lymphocyte populations is increasingly recognized as critical. Th1 cells, pivotal through interferon-gamma (IFN-γ) production, act alongside functional counterparts like Th17 cells and Th1–Th17 hybrid populations, which demonstrate complementary roles in host defense (7, 8). Concurrently, innate and innate-like immune cells are significant for differentiating infection stages. Neutrophils dominate airway infiltrates in patients with ATB (9), playing dual roles in bacterial clearance and immunopathology (10). Natural killer (NK)-cell dynamics also reflect disease progression, with a progressive depletion of the CD3^−^CD7^+^GZMB^+^ NK subset observed from healthy controls through ATB, followed by post-treatment recovery (11, 12).

Given this circulating cellular heterogeneity, comprehensive immunophenotyping through surface marker analysis offers valuable insights for biomarker discovery. Our systematic investigation evaluates multiple immune populations implicated in anti-mycobacterial responses, including CD4^+^/CD8^+^ T cells, γδ T cells, NKT cells, NK cells, and mucosal-associated invariant T (MAIT) cells (identified by TCR Vα7.2 expression) (13). Critically, we extend this characterization to B-cell subsets, whose roles in TB pathogenesis have been historically underappreciated despite emerging evidence of their immunological significance.

The conventional paradigm viewed B cells in intracellular pathogen defense primarily through the lens of humoral immunity and immunoglobulin production (14). However, contemporary research reveals multifaceted roles for B cells, including antigen presentation, cytokine modulation, and T-cell regulation (15–17). B cells can internalize mycobacteria via receptor-mediated uptake and macropinocytosis, facilitating MHC class II antigen presentation. Mirroring T-cell diversity, B-cell populations demonstrate functional specialization through distinct subpopulations: naïve B cells (Bn), transitional B cells (Btr), antibody-secreting cells (ASCs), and memory B cells (MBCs) (18, 19).

Current literature, however, presents conflicting reports on peripheral B-cell dynamics during TB progression. Comparative analyses of ATB versus healthy controls describe unchanged (20), elevated (21), or diminished (22, 23) total B-cell frequencies. Similarly, contradictory patterns emerge in LTBI cohorts showing decreased B-cell counts (22), while post-treatment patients exhibit B-cell expansion (20). This ambiguity highlights the need for a more refined analysis of B-cell subset distribution and differentiation across the TB disease spectrum.

In this study, we performed multiparameter flow cytometric profiling of peripheral blood immune cells from patients with MDR-TB, individuals with LTBI, and healthy controls. We aimed to characterize broad immune alterations with particular emphasis on B-cell subset distribution, while carefully acknowledging the limitations inherent to peripheral blood-based and cross-sectional analyses.

## Materials and methods

### Study population and ethical considerations

This study was approved by the ethics committee of the affiliated hospital of Zunyi Medical University, China (No. KLL-2022-579). Individuals with ATB, LTBI (IGRA^+^), and healthy controls (IGRA^−^) were recruited from the Affiliated Hospital of Zunyi Medical University. IGRA^+^ and IGRA^−^ participants were recruited from hospital staff undergoing routine health screening and community volunteers without TB-related symptoms. Asymptomatic individuals with positive IGRA results were confirmed by TB-IGRA (Beijing Wantai Biological Pharmacy Enterprise Co., Ltd.). Patients with ATB were microbiologically confirmed and, in this cohort, all ATB cases had drug-resistant TB and were receiving second-line anti-TB treatment at the time of sampling. This characteristic of the cohort is acknowledged as a limitation and may affect the generalizability of the findings to drug-sensitive TB.

The sex distribution of the enrolled cohorts, particularly the male predominance in the ATB group, reflects the established epidemiological profile of TB, where global incidence is consistently higher in male patients. This disparity is representative of the local clinical population from which participants were recruited.

### PBMC isolation and processing

Peripheral blood samples (8–10 mL per participant) were collected in sodium heparin vacutainers (BD Biosciences) and processed within 2 h to ensure cell viability. Peripheral blood mononuclear cells (PBMCs) were isolated using Ficoll-Hypaque (GE Healthcare) density gradient centrifugation at 400 × *g* for 30 min at room temperature. The PBMC layer was carefully aspirated, washed twice with phosphate-buffered saline (PBS, Gibco), and resuspended in complete RPMI 1640 medium (Corning) supplemented with 10% heat-inactivated fetal bovine serum (FBS, Gibco).

### Multicolor flow cytometry analysis

Surface staining: PBMCs were thawed and washed. The staining and gating strategy was adapted from previously published phenotypic analyses in TB (18). In brief, a total of 1 × 10^6^ cells were stained with a pre-optimized antibody panel (details and clones provided in **Supplementary Table S1**) in Brilliant Stain Buffer (BD Biosciences) for 30 min at 4 °C in the dark.

Data acquisition and analysis: Stained cells were acquired on a Cytek Aurora spectral flow cytometer (Cytek Biosciences) configured with five lasers (355, 405, 488, 561, and 640 nm). Compensation was performed using single-stained UltraComp eBeads (Thermo Fisher Scientific). Gating strategies for B-cell subpopulations (based on CD27, IgD, and CD10 expression) were established using Fluorescence Minus One (FMO) controls, as depicted in **Supplementary Figure S2**. Data were analyzed using FlowJo v10.7.1 (BD Biosciences). For visualization, high-dimensional data were subjected to dimensionality reduction using the Uniform Manifold Approximation and Projection (tSNE) algorithm in FCS Express™ 7 Software (Denovo Software). Cell populations were quantified as percentages of parent populations (%).

### Interferon gamma release assays

The IGRA test was employed to evaluate the TB infection status of all participants. IGRA was performed according to the manufacturer’s instructions (Wantai, Beijing, China). Briefly, blood samples were obtained and divided into three tubes: (1) a negative control tube (Nil tube), (2) an *Mtb*-specific antigen stimulation tube (Test tube), and (3) a positive control tube containing PHA (PHA tube). After incubation at 37°C for approximately 22 h, the supernatants were collected, and IFN-γ was quantitated by immunoassays. IGRA results were judged according to the manufacturer’s instruction.

### Statistical analysis

Continuous variables are presented as mean ± standard deviation (SD) for normally distributed data or median [interquartile range (IQR)] for non-parametric data. Normality was assessed via Shapiro–Wilk test. The following group comparisons were employed:

Parametric: Student’s *t*-test (two groups) or one-way analysis of variance (ANOVA) with Tukey’s *post-hoc* test (≥3 groups).

Non-parametric: Mann–Whitney *U* test (two groups) or Kruskal–Wallis test with Dunn’s correction (≥3 groups).

Categorical variables were analyzed using Fisher’s exact test or Pearson’s χ^2^ test with Yates’ continuity correction. Correlation analyses utilized Spearman’s rank correlation coefficient. All tests were two-tailed, with *p* < 0.05 considered statistically significant. Analyses were performed using GraphPad Prism v8.4.2 (GraphPad Software).

## Results

### Participant characteristics

Demographic and clinical characteristics of the participants are summarized in **Table 1**. The mean ages were comparable across the ATB, IGRA^+^, and IGRA^–^ groups, supporting the validity of group-wise immunological comparisons.

**Table 1:** Clinical characteristics of participants.

Factor	Characteristic	IGRA^−^	IGRA^+^	ATB
Sex	Male	2	2	7
	Female	6	6	1
Age	Years, mean (range)	43 (22–59)	45 (23–60)	49 (24–71)
Quantitative results	pg/mL (range)	1.5 (0–3.5)	186.1 (14–500.8)	220.0 (16.5–471.8)

IGRA^−^: non-TB infected (*n* = 8); IGRA^+^: asymptomatic infected (*n* = 8); ATB: patients with active tuberculosis (*n* = 8).

### Comprehensive immune cell profiling of peripheral blood subsets via flow cytometry

**Figure 1:**
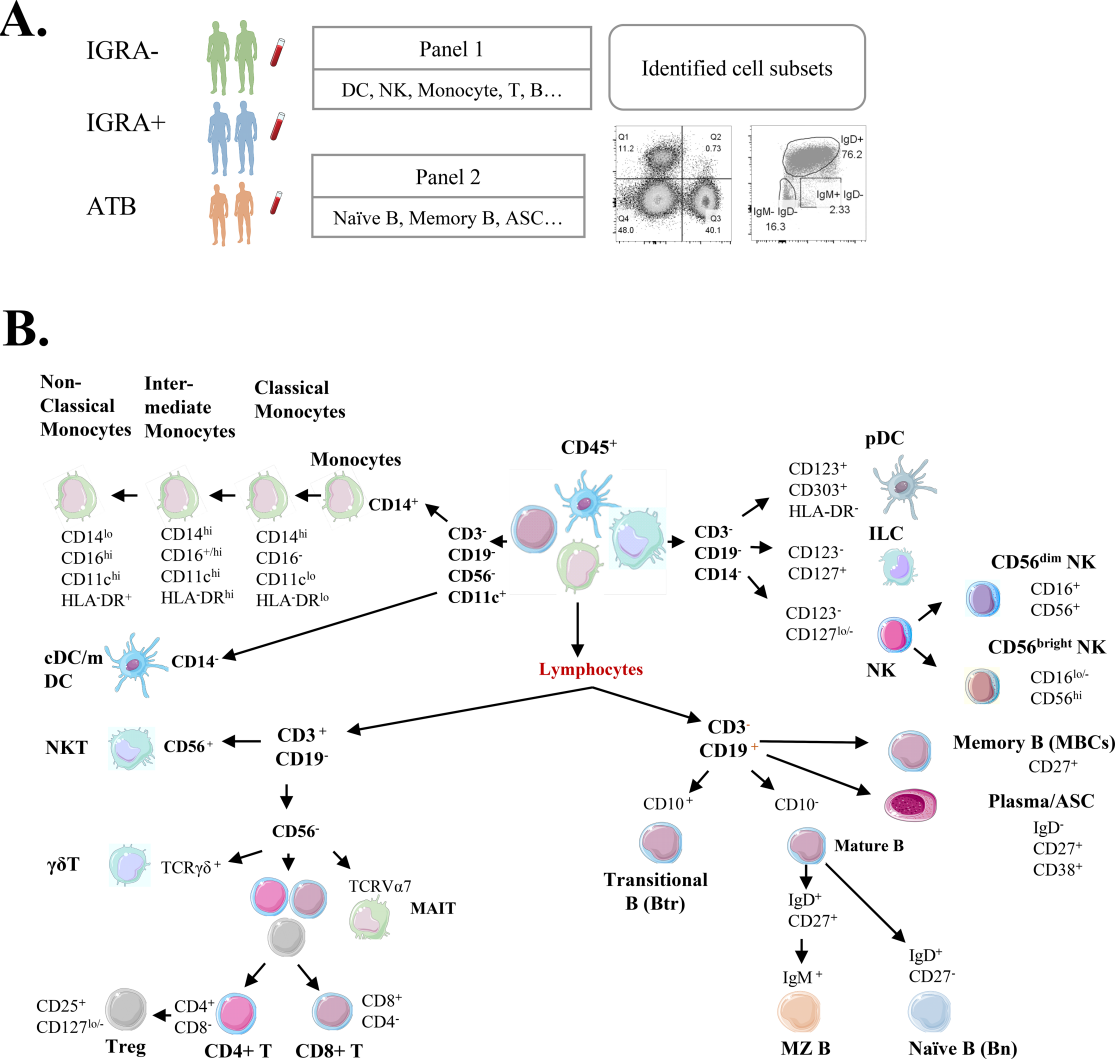
Study workflow and immune phenotyping of human PBMCs. **(A)** Schematic overview of the experimental procedure. PBMCs were isolated from all participants across the three cohorts (active tuberculosis, ATB; latent TB infection, IGRA^+^; and healthy controls, IGRA–). After cryopreservation, PBMCs were thawed and subjected to high-dimensional immunophenotyping using a predefined 21-marker panel by flow cytometry (including 16 markers in Panel 1 and 8 markers in Panel 2). The resulting high-parameter data were analyzed to identify immune cell populations and states associated with TB disease status. **(B)** Representative flow cytometry plots illustrating the sequential gating strategy used to identify major immune cell populations and subsets. The gating hierarchy began with the identification of single, live lymphocytes based on forward-scatter (FSC) and side-scatter (SSC) properties, followed by successive steps to delineate innate and adaptive immune subsets. Eighteen immune cell populations identified in Panel 1 include CD19^+^CD3^-^ B cells, CD3^+^CD19^-^ T cells, plasmacytoid dendritic cells (pDCs), conventional/myeloid dendritic cells (cDCs/mDCs), CD4^+^ T cells, CD8^+^ T cells, regulatory T cells (Treg), natural killer T (NKT) cells, γδ T cells, mucosal-associated invariant T (MAIT) cells, innate lymphoid cells (ILC), NK cells (including CD56^bright^ and CD56^dim^ subsets), and monocyte subsets (classical, intermediate, and non-classical monocytes). B-cell subsets shown in Panel 2 include memory B cells (MBCs), antibody-secreting cells (plasma/ASCs), naïve B cells (Bn), circulating marginal zone–like B cells (MZ B), and transitional B cells (Btr). This systematic approach ensured consistent and reproducible subset discrimination across all samples.

A total of 24 participants were enrolled in this study and classified into three distinct groups: 8 patients with ATB, 8 individuals with LTBI (IGRA^+^), and 8 healthy controls (IGRA^–^). PBMCs were isolated from each participant and cryopreserved for subsequent high-dimensional immunophenotyping by flow cytometry (**Figure 1A**). All samples met quality thresholds for cell count and viability to ensure robustness in downstream analyses.

To delineate immune dynamics in ATB, we designed a multiparameter flow cytometry panel incorporating the following markers: CD45, CD3, CD19, CD56, CD11c, CD14, CD16, HLA-DR, CD123, CD303, CD127, CD25, CD4, CD8, TCRγδ, TCRVα7.2, CD10, IgD, CD27, CD38, and IgM. This panel enabled the identification of major immune lineages and subsets (**Figure 1B**).

Leukocyte populations were gated via CD45 expression and stratified into lymphoid (CD3^+^ T cells, CD19^+^ B cells, CD3^+^CD56^+^ NKT-like cells) and myeloid lineages. T-cell subsets were defined as CD4^+^/CD8^+^ subsets. Specialized T populations: MAIT cells (TCR Vα7.2^+^) and regulatory T cells (Tregs: CD25^+^CD127^low^CD4^+^).

Myeloid compartment analysis from CD3^−^CD19^−^ cells included the following: Monocyte subsets: classical (CD14^+^CD16^−^), intermediate (CD14^+^CD16^+^), and nonclassical (CD14^dim^CD16^+^). NK cell stratification: cytotoxic CD56^dim^CD16^high^ vs. cytokine-secreting CD56^bright^CD16^low^ subsets, with activation status assessed via HLA-DR expression. Dendritic cells (DCs): Identified as HLA-DR^+^CD11c^+^CD16^−^.

To ensure cross-sample comparability, cryopreserved PBMCs from patients with ATB, IGRA^+^, and IGRA^−^ cohorts were thawed and analyzed synchronously within identical experimental runs, minimizing batch variability.

### General immune cell profiling via flow cytometry

**Figure 2:**
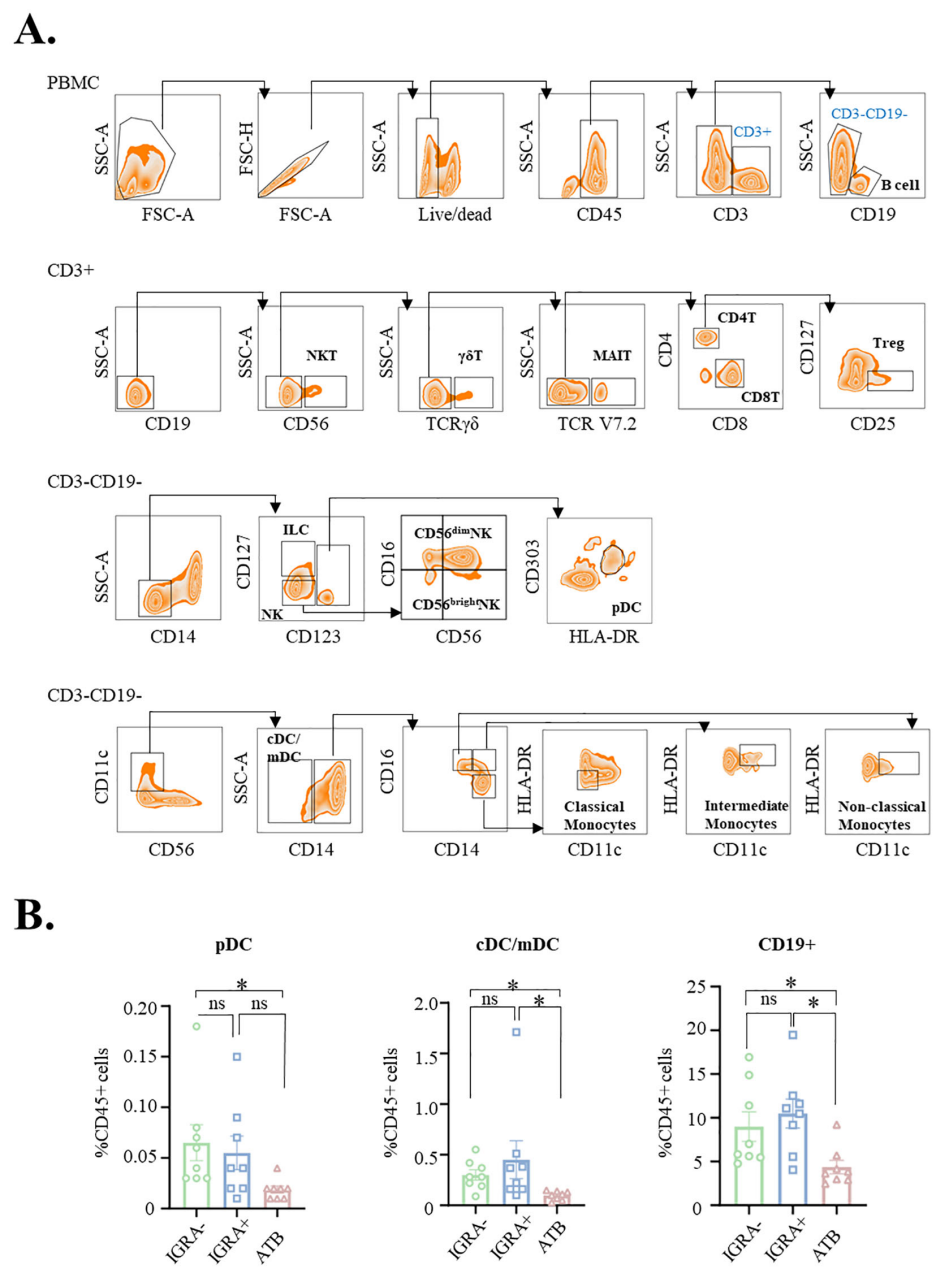
Alterations in circulating immune cell populations in active tuberculosis. **(A)** Gating strategy for the identification of major immune cell types, including T cells (CD4^+^, CD8^+^, and Tregs), NK cells, monocytes, and B cells, using the 16-color flow cytometry panel (Panel 1). **(B)** Comparative frequencies of dendritic cell (DC) subsets [plasmacytoid DCs (pDCs) and conventional DCs (cDCs)] and total CD19^+^ B cells among the three participant groups: active tuberculosis (ATB), latent tuberculosis infection (IGRA^+^), and healthy controls (IGRA^−^). Data are presented as mean ± SD. Statistical significance was determined by one-way ANOVA; *p < 0.05.

To characterize the immune landscape in ATB, we used a 16-color flow cytometry panel (Panel 1) to evaluate the frequencies of major immune cell types, including T cells, NK cells, monocytes, and B cells (**Figure 2A**). Gating was performed hierarchically: B cells were identified as CD19^+^CD3^-^ lymphocytes. Among CD19^-^CD3^+^ T-lineage cells, NKT cells were defined as CD3^+^CD56^+^, while the remaining CD3^+^CD56^-^ cells were further separated into γδ T cells (TCRγδ^+^) and TCRγδ^-^ conventional T cells. From the latter, MAIT cells were gated as TCRVα7.2^+^, and the remaining TCRVα7.2^-^ conventional T cells were subdivided into CD4^+^ T cells (CD4^+^CD8^-^) and CD8^+^ T cells (CD4^-^CD8^+^). Regulatory T cells (Tregs) were subsequently identified as CD25^+^CD127^-^ within the CD4^+^ T-cell population.

Within the CD3^-^CD19^-^ compartment, innate lymphoid cells (ILCs) were defined as CD127^+^CD123^-^, and NK cells were gated as CD127^-^CD123^-^CD56^+^ and further stratified into CD56^bright^ (CD16^-^CD56^+^) and CD56^dim^ (CD16^+^CD56^+^) subsets. Plasmacytoid dendritic cells (pDCs) were identified as CD123^+^CD303^+^HLA-DR^+^. From the CD11c^+^ fraction of the CD3^-^CD19^-^ lineage, conventional dendritic cells (cDCs) were defined as CD11c^+^CD14^-^, and monocyte subsets were classified as follows: classical monocytes (CD14^high^CD16^low^), intermediate monocytes (CD14^high^CD16^high^CD11c^high^HLA^−^DR^high^), and non-classical monocytes (CD14^low^CD16^high^CD11c^high^HLA^−^DR^+^).

This gating strategy enabled consistent and reproducible identification of 18 distinct immune cell subsets, facilitating the analysis of their compositional changes across clinical groups. Consistent with previous reports (24, 25), we observed no significant differences in the frequencies of CD4^+^ T, CD8^+^ T, or regulatory T cells (Tregs) between patients with ATB and healthy controls (IGRA^–^). Similarly, NK cell counts remained comparable across groups, suggesting that both circulating cellular adaptive and innate effector populations are largely maintained during active disease (**Supplementary Figure S1**). However, Supplementary **Figure 1** reveals significant alterations in specific innate-like lymphocyte populations. Notably, both patients with ATB and IGRA^+^ individuals exhibited a reduction in circulating γδ T cells compared to IGRA^–^ controls, with the difference reaching statistical significance for patients with ATB. Additionally, MAIT cell frequencies were significantly lower in patients with ATB than in IGRA^–^ individuals.

In contrast, DCs—including both plasmacytoid (pDC) and conventional/myeloid (cDC/mDC) subsets—were significantly reduced in patients with ATB compared to both IGRA^+^ and IGRA– groups (**Figure 2B**). Most notably, we observed a striking reduction in total peripheral B cells (CD19^+^) in patients with ATB relative to both IGRA^+^ individuals and healthy controls (*p* < 0.05; **Figure 2B**). This finding is consistent with growing evidence of B-cell dysregulation in TB. Depletion of circulating B cells may reflect impaired humoral immune function, affecting key mechanisms such as antibody production, antigen presentation to T cells, and immunomodulatory cytokine signaling. The observed B-cell deficit suggests a broad disruption of adaptive immunity that could facilitate *Mtb* persistence by undermining coordinated immune responses.

### B-cell maturation arrest and functional impairment in ATB

B-cell development begins in the bone marrow, where immature B cells are generated and then enter the peripheral blood. These immature B cells proceed through several transitional stages, named transitional B cells (Btr), before migrating to secondary lymphoid organs, such as the spleen. In the spleen, B cells undergo rigorous antigen selection, which influences their differentiation into mature B cells, including MBCs and plasma cells, which are both involved in the adaptive immune response.

**Figure 3:**
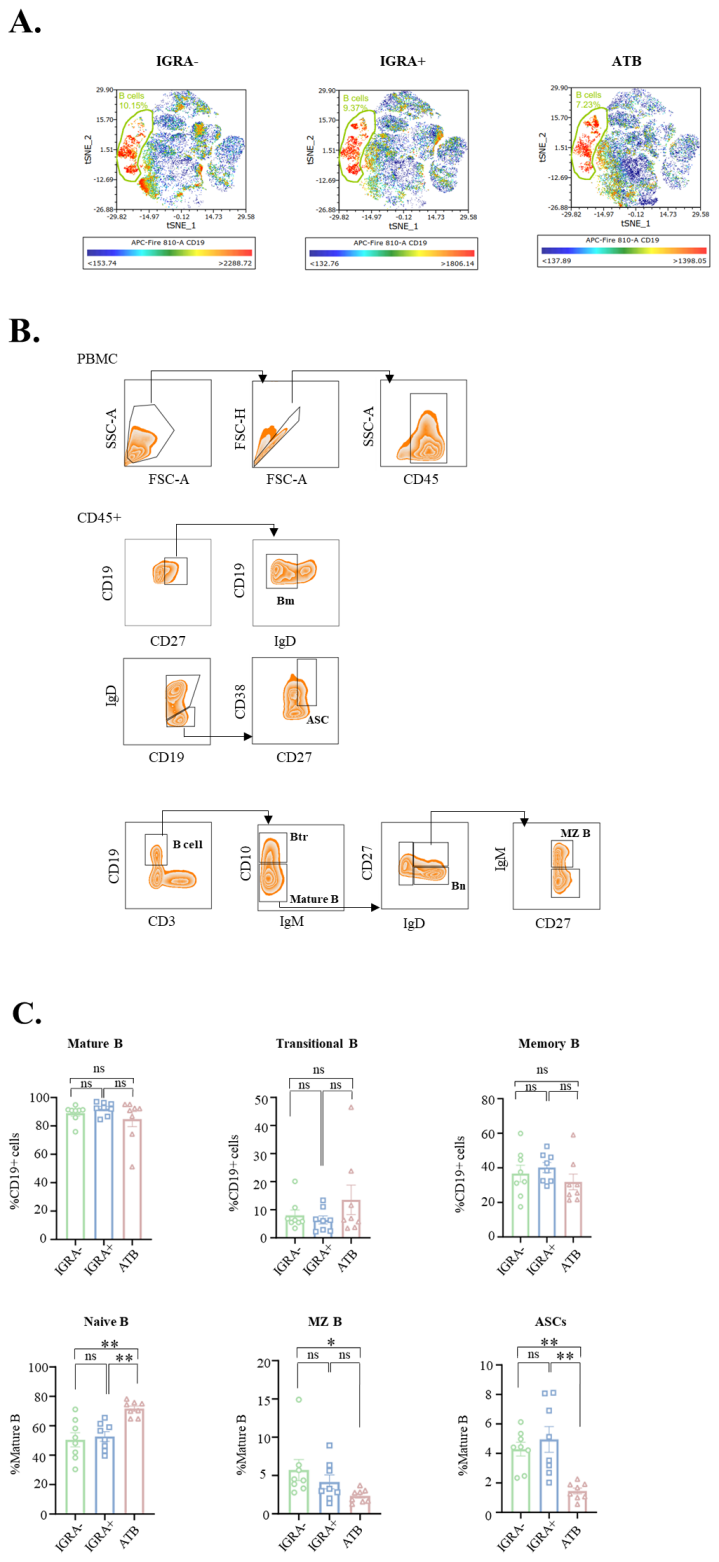
Distribution of major circulating B-cell subsets in peripheral blood from IGRA^−^ (n = 8), IGRA^+^ (n = 8) and patients with ATB (n = 8). **(A)** t-SNE visualization of PBMCs with cells colored according to CD19 expression levels. CD19^+^ B cell populations are highlighted with green ellipses in the IGRA^−^, IGRA^+^, and ATB groups. **(B)** After lymphocyte gate, the CD19^+^ (B cell) population gates and B-cell subsets were identified by distinct surface markers. **(C)** Frequencies of total B cells (CD19^+^) as % of live lymphocytes, transitional B cells (CD19^+^CD10^+^), and mature B cells (CD19^+^CD10^−^) as % of total B cells, naïve B cells (CD19^+^CD10^−^IgD^+^CD27^−^), MZ B cells (CD19^+^CD10^−^IgD^+^CD27^+^), and ASCs (CD19^+^IgD^−^CD27^+^CD38^+^) as % of mature B cells. Statistical analyses were done by one-way ANOVA test. Significant differences are shown on top of the respective columns (*p* < 0.05; *p < 0.01). ns, not significant.

Commonly, in the peripheral blood, B-cell ontogeny involves sequential maturation from bone marrow-derived transitional B cells (Btr: CD10^+^) to splenic naïve B cells (Bn: IgD^+^CD27^−^), followed by antigen-driven differentiation into MBCs (IgD^−^CD27^+^) and ASCs (CD19^+^CD38^high^) (18). Flow cytometry profiling (Panel 2: CD45/CD3/CD19/CD10/IgM/IgD/CD27/CD38) revealed profound perturbations in ATB: expanded naïve B cells (Bn: +40% vs. controls, *p* < 0.01), depleted MZ B cells (−59%, *p* < 0.05), and ASCs (−65%, *p* < 0.01) (**Figures 3A-C**). The significant expansion of Bn concurrent with a severe reduction in ASCs and MZ B cells indicates a profound B-cell maturation arrest, likely resulting from *Mtb*-induced suppression of germinal center formation or impaired antigen-presenting cell function. The paucity of ASCs further indicates compromised antibody production capacity, which may undermine opsonization and complement activation against extracellular bacilli.

### Reduction in circulating marginal zone B cells serves as a diagnostic indicator for active tuberculosis

**Figure 4:**
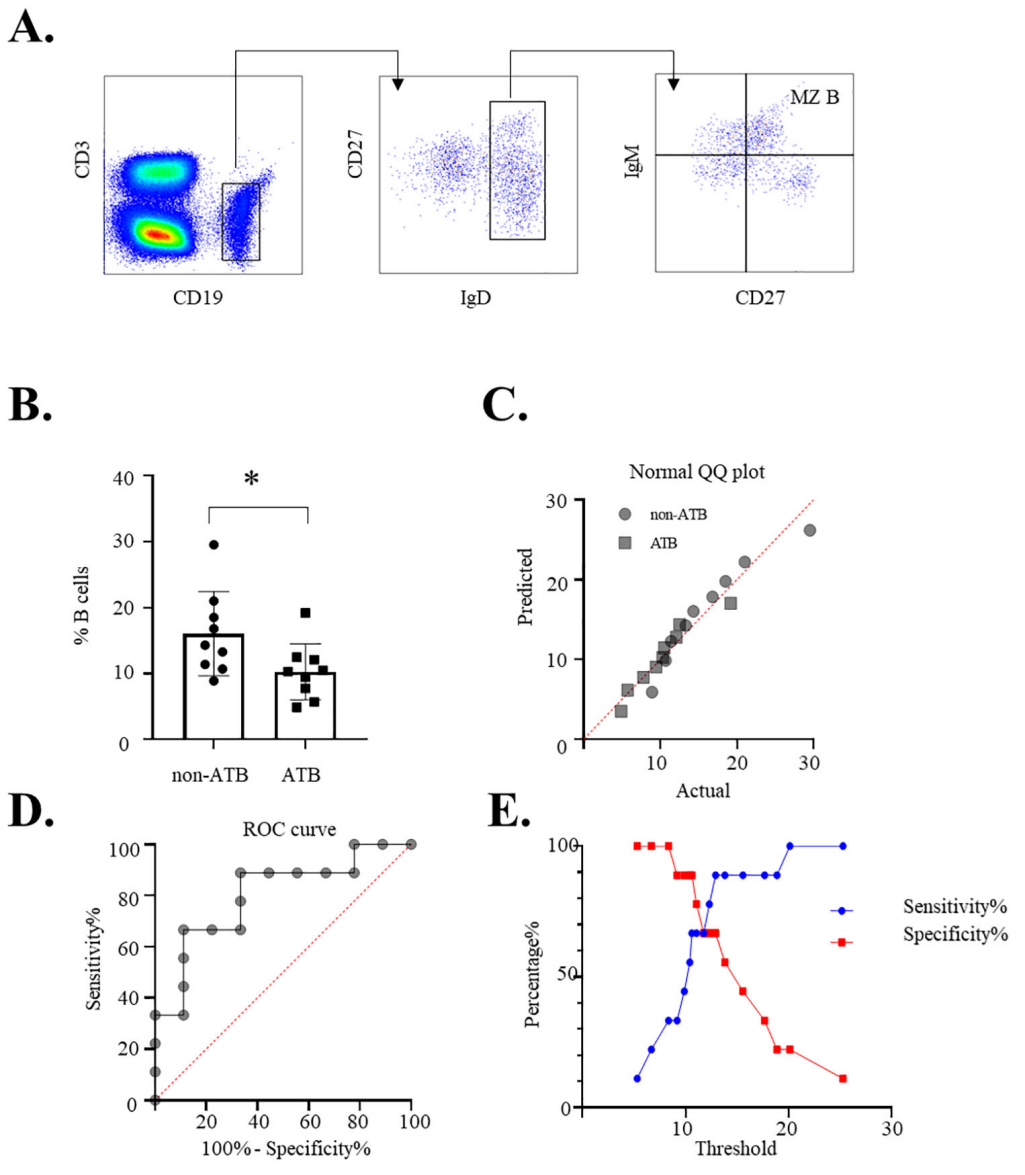
Decreased frequency of circulating marginal zone-like B cells in active tuberculosis and its diagnostic performance. **(A)** Gating strategy for the identification of human MZ B cells. Peripheral blood mononuclear cells were stained with a panel of antibodies (CD3, CD19, CD27, IgD, and IgM). After gating on lymphocytes and excluding CD3^+^ T cells, B cells were identified as CD19^+^ lymphocytes. MZ B cells were defined within the CD19^+^ B-cell population as IgD^+^CD27^+^IgM^+^. **(B)** Comparison of circulating MZ B-cell frequencies between ATB and non-ATB groups. MZ B-cell frequency is expressed as a percentage of total CD19^+^ B cells. Statistical differences between groups were analyzed using an unpaired t-test. **(C)** Normality of data distribution for the frequency of MZ B cells (as a percentage of total B cells) in the ATB and non-ATB groups was confirmed using Anderson–Darling, D’Agostino and Pearson, Shapiro–Wilk, and Kolmogorov–Smirnov tests (all p > 0.05). **(D)** Receiver operating characteristic (ROC) curve analysis evaluating the diagnostic performance of the MZ B-cell frequency for discriminating patients with ATB from non-ATB controls. The analysis resulted in an area under the curve (AUC) of 0.8025 (95% CI: 0.5915 to 1.000, p = 0.0305). **(E)** Sensitivity and specificity as a function of the classification threshold for MZ B-cell frequency.

Building upon our previous findings of a generalized B-cell maturation arrest and functional impairment in ATB, we sought to identify a specific and clinically measurable B-cell subset alteration. To this end, we developed a targeted five-color flow cytometry panel (CD3, CD19, CD27, IgD, and IgM) for practical immunophenotyping. After gating on lymphocytes and identifying CD19^+^ B cells (using CD3 for exclusion), we defined MZ B cells as the IgD^+^CD27^+^IgM^+^ population (**Figure 4A**). Strikingly, the proportion of this specific MZ B subset within total B cells was significantly contracted in patients with ATB compared to the non-ATB control group (**Figure 4B**). This control cohort included individuals with LTBI, closely exposed healthcare workers, and patients with other pulmonary diseases, strengthening the specificity of our observation. This finding suggests that the loss of circulating MZ B cells is a specific feature of active disease, not merely a consequence of recent exposure or general pulmonary inflammation.

The data for both groups passed all tests for normality (Anderson-Darling, D’Agostino and Pearson, Shapiro–Wilk, and Kolmogorov–Smirnov; all *p* > 0.05) (**Figure 4C**), validating the use of parametric statistical tests. Most importantly, receiver operating characteristic (ROC) curve analysis demonstrated that this simple MZ B immunophenotype held significant diagnostic power. The analysis yielded an area under the curve (AUC) of 0.8025 (95% CI: 0.5915 to 1.000, *p* = 0.0305) (**Figure 4D**), indicating good utility in discriminating ATB from non-ATB conditions. In addition, analysis of sensitivity and specificity across varying classification thresholds further illustrated the performance characteristics of MZ B-cell frequency as a diagnostic marker (**Figure 4E**). Therefore, the quantifiable reduction in MZ B cells provides a tangible and translatable biomarker. The ability to capture this aspect of the dysfunctional B-cell response using a minimal, clinically feasible antibody panel highlights its utility for immunodiagnostic application in ATB.

## Discussion

This study offers valuable insights into the immune dysregulation observed in MDR-TB, with a particular emphasis on B-cell alterations. Our findings reveal a significant reduction in total B cells, alongside a marked depletion of ASCs in the peripheral blood of patients with MDR-TB, which may contribute to the persistence and chronicity of *Mtb* infection (26, 27).

Additionally, the depletion of MZ B cells, which are critical for rapid immune responses to encapsulated pathogens, further implicates B-cell dysfunction in the pathogenesis of TB. Recent studies have reported the important role of MZ B cells in the lungs, the primary organ affected by TB, in orchestrating effective immune responses (28, 29). In our study, we also observed a close relationship between MZ B cells and *Mtb* infection in peripheral blood, suggesting that these cells may continue to play a role throughout the course of infection. Furthermore, the use of peripheral blood as a readily accessible source for studying MZ B cells has important diagnostic implications, given the challenges in obtaining lung tissue from patients with ATB.

These findings challenge the traditional T cell-centric view of TB immunopathology. While our data confirm preserved T/NK-cell frequencies in ATB (consistent with prior reports), the concurrent collapse of humoral effector mechanisms—naïve B-cell accumulation, ASC deficiency, and MZ B-cell depletion—implies that *Mtb* subverts multiple immune axes. This aligns with growing recognition of B cells as active participants in TB defense through antibody-independent roles like cytokine modulation and lymphoid follicle organization (15–17).

Future studies should address whether MZ B-cell depletion directly enables bacterial dissemination or merely marks advanced disease. Longitudinal tracking of this subset during treatment could clarify its biomarker utility, while spatial transcriptomics of granulomas may resolve the “missing MZ B cell” paradox.

Our study acknowledges several inherent limitations. First, the modest cohort size and the predominance of male patients in the ATB group curtail statistical power and may affect the generalizability of the results. Second, the focus exclusively on patients with MDR-TB means the findings may not be directly applicable to those with drug-sensitive TB, reflecting a more severe immunopathological state. Third, from a methodological perspective, the use of peripheral blood as a proxy for systemic immunity, though pragmatic, carries constraints. The absence of paired tissue samples from sites such as the lung, spleen, or bone marrow prevents definitive characterization of MZ B-cell trafficking and leaves unresolved the functional competence (e.g., IgM secretion) of the circulating MZ B cells we identified.

## Data Availability

The original contributions presented in the study are included in the article/**Supplementary Material**. Further inquiries can be directed to the corresponding authors.

## Supplementary material

Supplementary Figure 1 Comprehensive analysis of innate and adaptive immune cell subsets across study groups. Frequencies of various immune cell populations within PBMCs are shown for the active tuberculosis (ATB), latent tuberculosis (IGRA^+^), and healthy control (IGRA^-^) groups. Analyzed subsets include: monocytes (classical [CD14^+^CD16^-^], intermediate [CD14^+^CD16^+^], non-classical [CD14^+^CD16^++^]); innate lymphoid cells (ILCs); NK cells (CD56^bright^ and CD56^dim^ subsets); NKT cells; γδ T cells; and T cell subsets (Tregs, CD4^+^ T, CD8^+^ T, and MAIT cells). Data are presented as mean ± SD. Statistical significance was determined by one-way ANOVA with appropriate *post-hoc* tests; **p* < 0.05, ns = not significant.

Supplementary Figure 2 Flow cytometric analysis of B-cell subpopulations based on CD27, IgD, and CD10 expression across study groups. In each plot, the Fluorescence Minus One (FMO) control (left column) is shown with a defined FMO Gate (dashed rectangle or polygon). This gate sets the threshold for positive vs. negative populations for the respective y-axis marker.

Supplementary Table 1 Antibody panel for spectral flow cytometry.
