# Supplementary material for: Peripheral blood B-cell compartment dysregulation in multidrug-resistant tuberculosis is associated with reduced circulating marginal zone-like B cells
Source: Front Immunol. 2026 Feb 10;17:1709981. doi: 10.3389/fimmu.2026.1709981 (PMC12929399; doi:10.3389/fimmu.2026.1709981)
Supplement: Supplementary Figure 1 — Comprehensive analysis of innate and adaptive immune cell subsets across study groups. Frequencies of various immune cell populations within PBMCs are shown for the active tuberculosis (ATB), latent tuberculosis (IGRA+), and healthy control (IGRA-) groups. Analyzed subsets include: monocytes (classical [CD14+CD16-], intermediate [CD14+CD16+], non-classical [CD14+CD16++]); innate lymphoid cells (ILCs); NK cells (CD56bright and CD56dim subsets); NKT cells; γδ T cells; and T cell subsets (Tregs, CD4+ T, CD8+ T, and MAIT cells). Data are presented as mean ± SD. Statistical significance was determined by one-way ANOVA with appropriate post-hoc tests; *p < 0.05, ns = not significant. [file DataSheet1.docx]

**Additional information**

Table S1. Antibody panel for spectral flow cytometry.

|  | Specificity | Fluorochrome | Clone | Order number | ul/test | Company |
| --- | --- | --- | --- | --- | --- | --- |
| 1 | CD45 | Spark NIR 685 | 2D1 | 368552 | 0.85 | BioLegend |
| 2 | CD3 | Alexa Fluor 700 | OKT3 | 317340 | 0.42 | BioLegend |
| 3 | CD19 | APC/Fire 810 | HIB19 | 302272 | 0.85 | BioLegend |
| 4 | CD56 | PE/Fire 700 | QA17A16 | 392428 | 1.7 | BioLegend |
| 5 | CD11c | BV785 | 3.9 | 301644 | 4 | BioLegend |
| 6 | CD14 | BV421 | MφP9 | 563743 | 0.85 | BD |
| 7 | CD16 | BUV563 | 3G8 | 568289 | 1 | BD |
| 8 | HLA-DR | PE/Fire 810 | L243 | 307683 | 0.85 | BioLegend |
| 9 | CD123 | Super Bright 702 | 6H6 | 67-1239-42 | 4 | eBioscience |
| 10 | CD303 | BV605 | 201A | 354224 | 4 | BioLegend |
| 11 | CD127 | RY586 | HIL-7R-M21 | 568139 | 4 | BD |
| 12 | CD25 | PE-Cy7 | M-A251 | 557741 | 1 | BD |
| 13 | CD4 | FITC | OKT4 | 317408 | 0.85 | BioLegend |
| 14 | CD8 | PerCP-eFluor 710 | OKT8 | 46-0086-42 | 0.8 | eBioscience |
| 15 | TCRγδ | APC | B1 | 555718 | 2 | BD |
| 16 | TCRVα7.2 | PE/Dazzle 594 | 3C10 | 351730 | 1.7 | BioLegend |
| 17 | CD10 | BV711 | HI10a | 312226 | 2 | BioLegend |
| 18 | IgD | BV421 | IA6-2 | 348226 | 1 | BioLegend |
| 19 | CD27 | BV510 | O323 | 302836 | 2.5 | BioLegend |
| 20 | CD38 | BV650 | HB-7 | 356620 | 1.5 | BioLegend |
| 21 | IgM | BUV737 | SA-DA4 | 367-9998-42 | 1.5 | eBioscience |
| 22 | Viability | FVD eFluor 780 | n/a | 65-0865-14 | 0.1 | eBioscience |


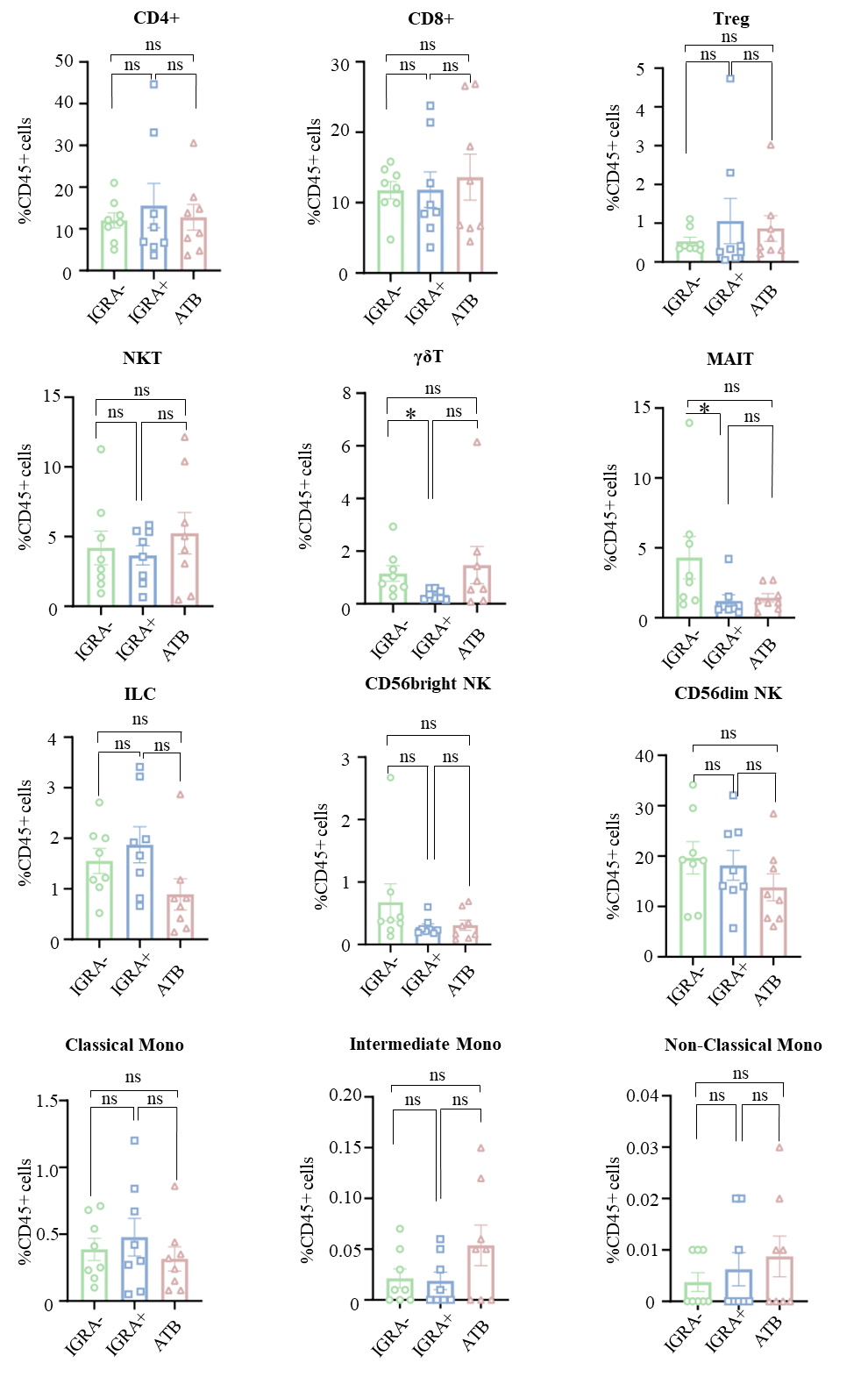


Figure S1. Comprehensive analysis of innate and adaptive immune cell subsets across study groups.

Frequencies of various immune cell populations within PBMCs are shown for the active tuberculosis (ATB), latent tuberculosis (IGRA⁺), and healthy control (IGRA⁻) groups. Analyzed subsets include: monocytes (classical [CD14⁺CD16⁻], intermediate [CD14⁺CD16⁺], non-classical [CD14^dim^CD16⁺]); innate lymphoid cells (ILCs); NK cells (CD56ᵇʳⁱᵍʰᵗ and CD56ᵈⁱᵐ subsets); NKT cells; γδ T cells; and T cell subsets (Tregs, CD4⁺ T, CD8⁺ T, and MAIT cells). Data are presented as mean ± SD. Statistical significance was determined by one-way ANOVA with appropriate post-hoc tests; *p < 0.05, ns = not significant.


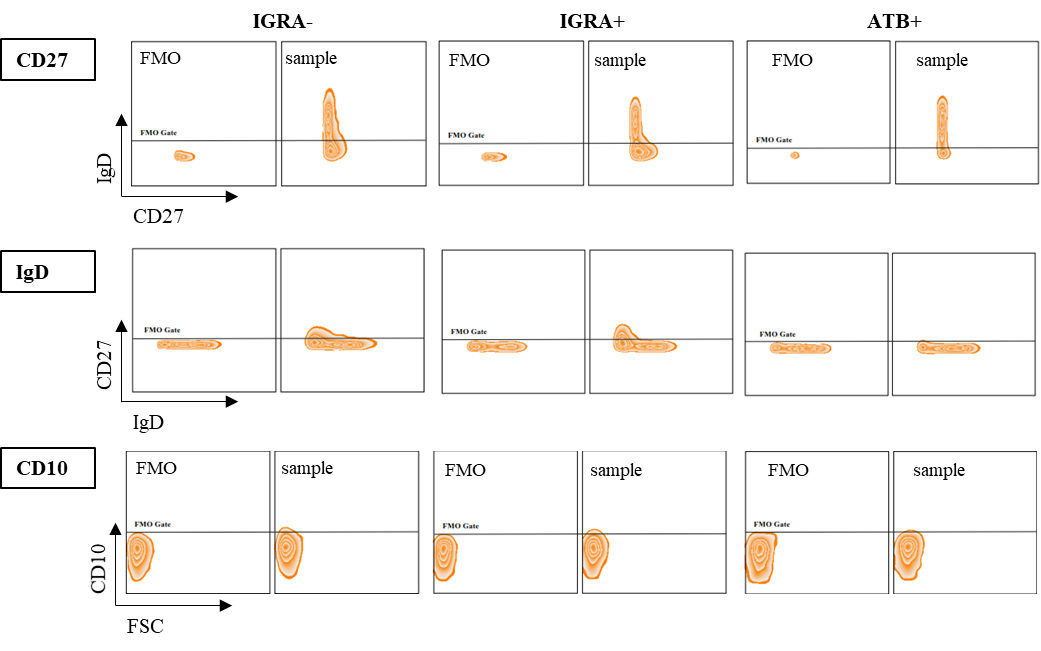


Figure S2. Flow cytometric analysis of B-cell subpopulations based on CD27, IgD, and CD10 expression across study groups. In each plot, the Fluorescence Minus One (FMO) control​ (left column) is shown with a defined FMO Gate​ (dashed rectangle or polygon). This gate sets the threshold for positive vs. negative populations for the respective y-axis marker.
